# Supplementary material for: Mitogenomes Reveal Two Major Influxes of Papuan Ancestry across Wallacea Following the Last Glacial Maximum and Austronesian Contact
Source: Genes (Basel). 2021 Jun 24;12(7):965. doi: 10.3390/genes12070965 (PMC8306604; doi:10.3390/genes12070965)
Supplement: Supplementary file 1 [file genes-12-00965-s001.zip › genes-1235426-supplementary/Supplemental FIgures S1-S4.pdf]

Supplementary Figures

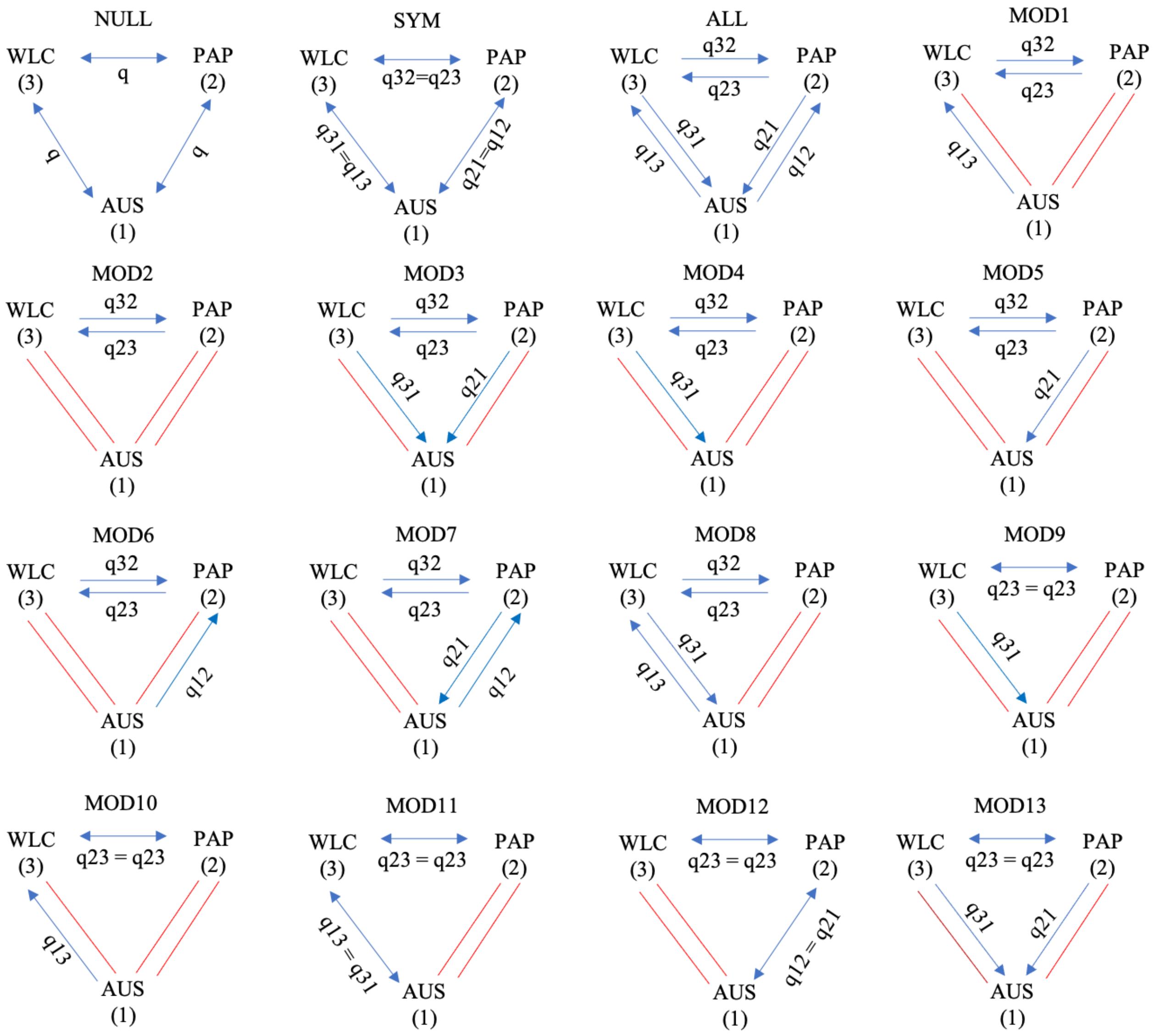

Figure S1. Complete set of migration models used in the BayesTraits analyses. 16 different migration models were tested for evidence of mtDNA lineage movements between all possible ordered pairs of regions: AUS = Australia; PAP = Papua (i.e. New Guinea and Island Melanesia); WLC = Wallacea. Model parameterisations ranged from independent migration rates between all of states (ALL) to a model where all rates were equal (NULL). All other models (SYM & MOD1 - MOD13) contained a mixture of independent (unidirectional blue arrows), dependent (bidirectional blue arrows) or constrained (i.e. no migration; red lines) migration paths.

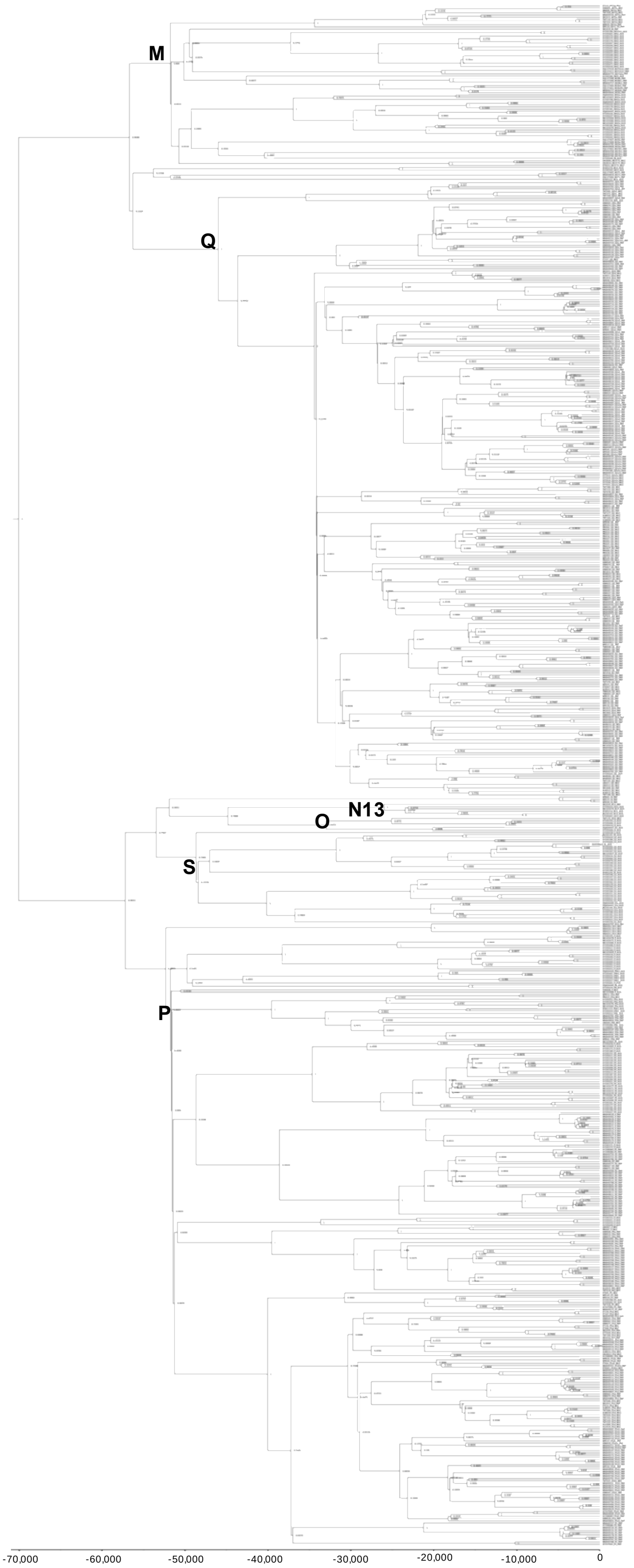

Figure S2. Phylogenetic tree displaying all 656 mitogenomes from Wallacea, New Guinea and Australia. Model parameters are listed in the Material and Methods section. Posterior support values are provided for all nodes.

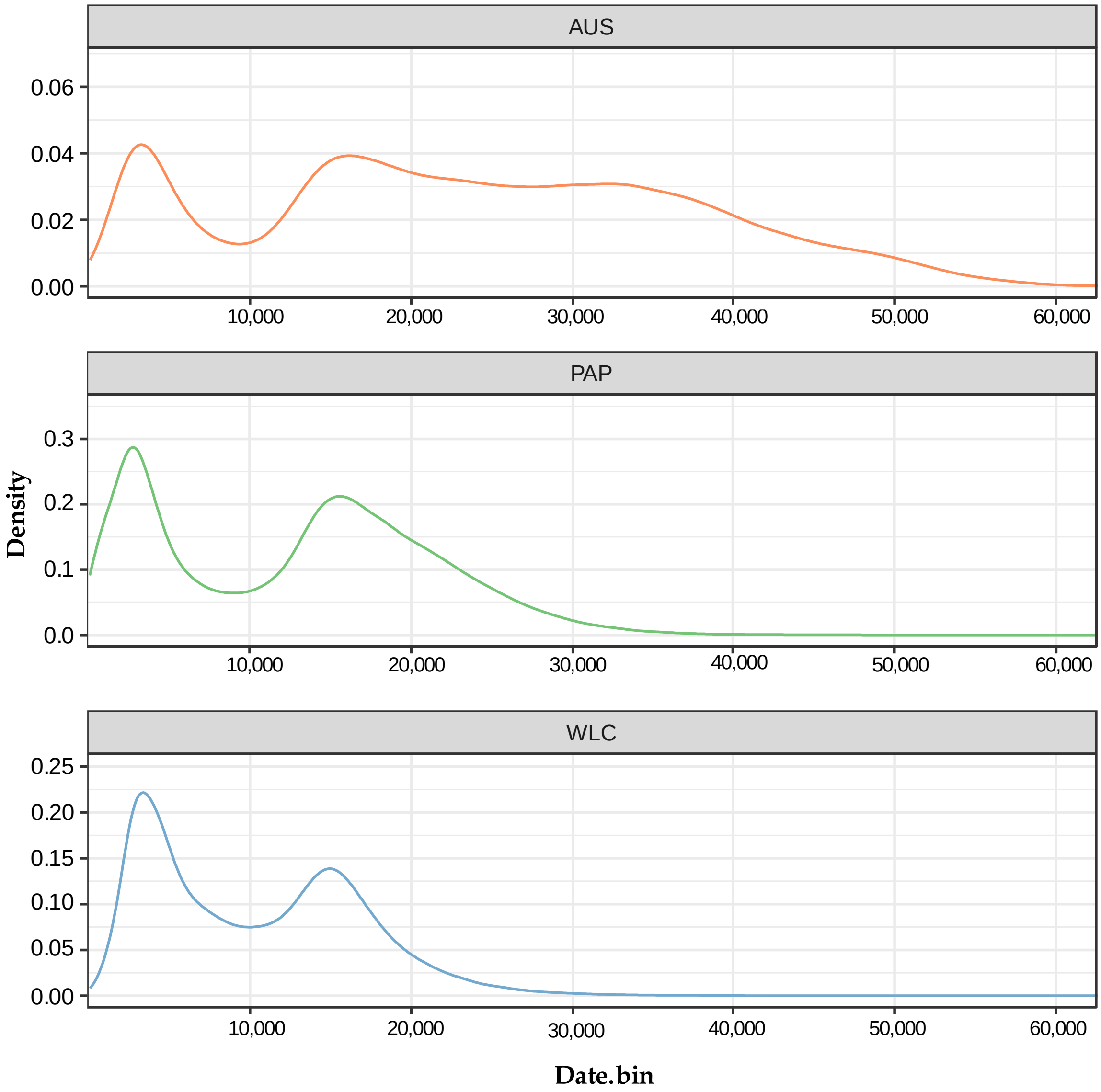

Figure S3. Distributions of TMRCAs across Wallacea, New Guinea, and Australia. TMRCAs were combined for all GECs in each of the three regions (shown in separate panels) and binned into ~100 year intervals to generate histograms. The kernel density estimates from figure 4A are replicated and shows a close match to the histograms, albeit underestimating the scale of the more recent cluster (though not the timing).

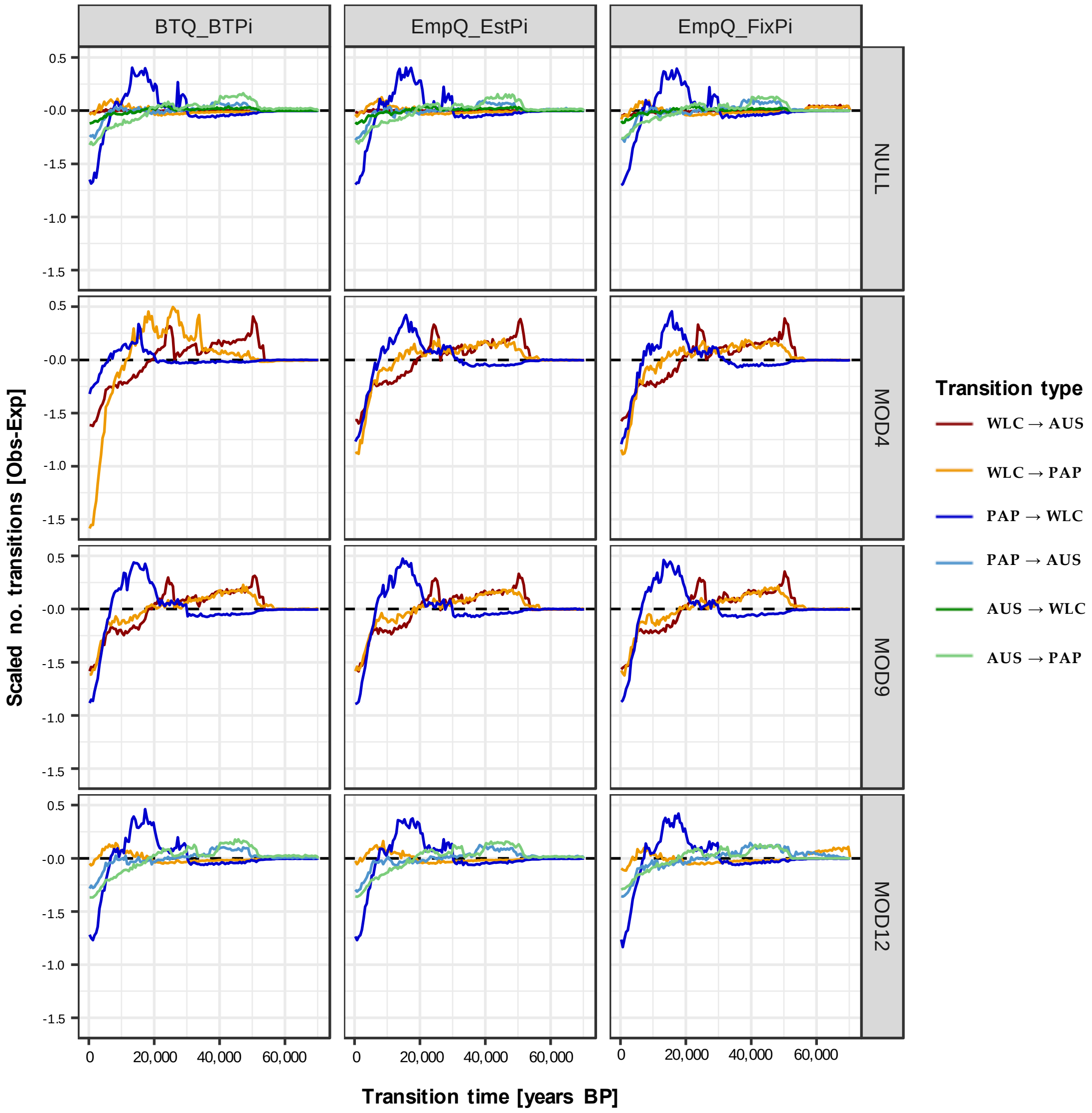

Figure S4. Estimated migration rates across Wallacea, New Guinea and Australia. Mean migration rates estimated from 1,000 SIMMAP stochastic mappings for the four best performing models inferred from BayesTraits (row panels). Migration rates (i.e. state transitions in stochastic mappings) are scaled to account for the expected number of migration events (see Materials and Methods). Three different parameter sets were used (column panels): BayesTraits estimates for the rate matrix and ancestral state (BTQ_BTPi), internally estimated rate matrix and equal ancestral states (EmpQ_EstPi) and internally estimated rate matrix and ancestral state fixed to Wallacea (EmpQ_FixPi).

Supplementary Tables

Table S1. Mitogenomes from Wallacea and West Papua. The 351 new mitogenomes used in this study, along with their associated population groupings, population IDs, haplogroup assignments, and informative polymorphic markers (see Methods 2.2) **.**

Table S2. Other published mitogenomes. The list of 470 publicly available mitogenomes and associated metadata from East Timor [1], New Guinea [2-4] and Australia [5-11] that were also used in our phylogeographic analyses. Haplogroups were re-assigned using same methods as for the newly reported mitogenomes (see Methods 2.2)

Table S3. Mitochondrial haplogroup frequencies. Population frequencies of each major haplogroup across the eight Wallacean and three West Papuan populations compared to the combined data from East Timor, New Guinea and Australia.

Table S4. TMRCAs for major haplogroups. TMRCAs estimated from the BEAST consensus tree. Haplogroup lineages associated with initial dispersal of AMH within Sahul (e.g. M42, P, Q, S) have credible intervals ranging between 40-60 kya based on a BEAST consensus tree using the mutation rate estimated from Posth et al [13].

Table S5. Geographically-exclusive clade (GEC) metadata. A total 68 GECs were extracted from the 10,000 posterior BEAST trees for Australian (AUS), Wallacean (WLC), and Papuan (PAP; i.e New Guinea and Island Melanesia) samples. For each GEC, we report the associated clade ID used in Fig. 3, along with the number of tips, median TMRCA, sampling region, and sample IDs.

Table S6. Summary of BayesTraits results. Log-transformed marginal likelihoods for model fit and the probabilities of the ancestral root state. More than half of the models (null, symmetrical rates (SYM), saturated (ALL), and models 1, 6, 7, 10, 11 and 12) support an Australian (AUS) ancestral state, with five models supporting Wallacea (WLC; models 2, 3, 4, 8 and 9) and two models supporting New Guinea (PAP; models 5 and 9) as the ancestral state.

Table S7. BayesTraits model comparison. Bayes Factors were calculated from the marginal likelihoods for all pairs of models (see methods 2.6.) Four best-fitting models were identified (i.e. the null model and models 4, 9 and 12) that differed from all other models but not each other at BF>2.

References

1. Gomes, S.M.; Bodner, M.; Souto, L.; Zimmermann, B.; Huber, G.; Strobl, C.; Röck, A.W.; Achilli, A.; Olivieri, A.; Torroni, A.; et al. Human Settlement History between Sunda and Sahul: A Focus on East Timor (Timor-Leste) and the Pleistocenic mtDNA Diversity. BMC Genomics 2015, 16, 70.
2. Merriwether, D.A.; Hodgson, J.A.; Friedlaender, F.R.; Allaby, R.; Cerchio, S.; Koki, G.; Friedlaender, J.S. Ancient mitochondrial M haplogroups identified in the Southwest Pacific. *Proc. Natl. Acad. Sci.* **2005**, *102*, 13034–13039, doi:10.1073/pnas.0506195102
3. Ingman, M.; Kaessmann, H.; Pääbo, S.; Gyllensten, U. Mitochondrial genome variation and the origin of modern humans. *Nature* **2000**, *408*, 708–713, doi:10.1038/35047064.
4. Pedro, N.; Brucato, N.; Fernandes, V.; André, M.; Saag, L.; Pomat, W.; Besse, C.; Boland, A.; Deleuze, J.-F.; Clarkson, C.; et al. Papuan Mitochondrial Genomes and the Settlement of Sahul. J. Hum. Genet. 2020, 65, 875–887.
5. van Holst Pellekaan, S.M.; Ingman, M.; Roberts-Thomson, J.; Harding, R.M. Mitochondrial genomics identifies major haplogroups in Aboriginal Australians. *Am. J. Phys. Anthropol.* **2006**, *131*, 282–294, doi:10.1002/ajpa.20426.
6. Friedlaender, J.S.; Friedlaender, F.R.; Hodgson, J.A.; Stoltz, M.; Koki, G.; Horvat, G.; Zhadanov, S.; Schurr, T.G.; Merriwether, D.A. Melanesian mtDNA Complexity. *PLoS One* **2007**, *2*, e248, doi:10.1371/journal.pone.0000248.
7. Hudjashov, G.; Kivisild, T.; Underhill, P.A.; Endicott, P.; Sanchez, J.J.; Lin, A.A.; Shen, P.; Oefner, P.; Renfrew, C.; Villems, R.; et al. Revealing the prehistoric settlement of Australia by Y chromosome and mtDNA analysis. *Proc. Natl. Acad. Sci. U. S. A.* **2007**, doi:10.1073/pnas.0702928104.
8. Heupink, T.H.; Subramanian, S.; Wright, J.L.; Endicott, P.; Westaway, M.C.; Huynen, L.; Parson, W.; Millar, C.D.; Willerslev, E.; Lambert, D.M. Ancient mtDNA sequences from the First Australians revisited. *Proc. Natl. Acad. Sci.* **2016**, *113*, 6892–6897, doi:10.1073/pnas.1521066113.
9. Rasmussen, M.; Guo, X.; Wang, Y.; Lohmueller, K.E.; Rasmussen, S.; Albrechtsen, A.; Skotte, L.; Lindgreen, S.; Metspalu, M.; Jombart, T.; et al. An Aboriginal Australian Genome Reveals Separate Human Dispersals into Asia. *Science (80-.).* **2011**, *334*, 94–98, doi:10.1126/science.1211177.
10. Nagle, N.; van Oven, M.; Wilcox, S.; van Holst Pellekaan, S.; Tyler-Smith, C.; Xue, Y.; Ballantyne, K.N.; Wilcox, L.; Papac, L.; Cooke, K.; et al. Aboriginal Australian mitochondrial genome variation – an increased understanding of population antiquity and diversity. *Sci. Rep.* **2017**, *7*, 43041, doi:10.1038/srep43041.
11. Wright, J.L.; Wasef, S.; Heupink, T.H.; Westaway, M.C.; Rasmussen, S.; Pardoe, C.; Fourmile, G.G.; Young, M.; Johnson, T.; Slade, J.; et al. Ancient nuclear genomes enable repatriation of Indigenous human remains. *Sci. Adv.* **2018**, *4*, 1–13, doi:10.1126/sciadv.aau5064.
12. Posth, C.; Renaud, G.; Mittnik, A.; Drucker, D.G.; Rougier, H.; Cupillard, C.; Valentin, F.; Thevenet, C.; Furtwängler, A.; Wißing, C.; et al. Pleistocene Mitochondrial Genomes Suggest a Single Major Dispersal of Non-Africans and a Late Glacial Population Turnover in Europe. Curr. Biol. 2016, 26, 827–833.
